# Genitourinary Tuberculosis, not Lupus

**DOI:** 10.1590/0037-8682-0571-2021

**Published:** 2022-04-08

**Authors:** Matheo Augusto Morandi Stumpf

**Affiliations:** 1 Universidade Estadual de Ponta Grossa, Ponta Grossa, PR, Brasil.

**FIGURE 1:**
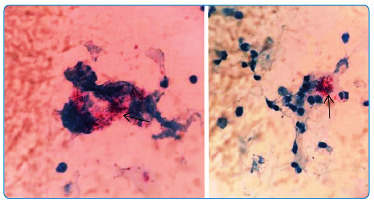
Ziehl-Neelsen stained acid-fast bacillus positive in the urine.

A 72-year-old woman presented with macroscopic hematuria for the past 2 weeks and weight loss within 6 months. She had cutaneous discoid lupus for 10 years and was taking hydroxychloroquine 400 mg daily. She denied having arthritis, dysuria, pollakiuria, or fever. She was initially diagnosed with lupus nephritis. The literature has well documented that some patients with cutaneous lupus may progress to systemic disease during follow-up^1^. However, admission examinations showed normal complement, negative antinuclear antibodies, elevated inflammatory markers, and no dysmorphic hematuria. The urine contained 22,000 leukocytes/mL and 80,000 erythrocytes/mL. Although she had no urinary symptoms, we collected a urine sample for culture, which was negative. On the third day of hospitalization, she had a vespertine fever (38 °C). Tuberculosis was suggested as a differential diagnosis when confronted with urine containing "sterile" leukocyturia. The Ziehl-Neelsen stained acid-fast bacillus (AFB) was positive in the urine (arrows in Figure 1), and *Mycobacterium tuberculosis* culture was positive. Computed tomography and ultrasonography of the genitourinary tract were normal. The patient was discharged on rifampicin, isoniazid, pyrazinamide, and ethambutol. Unfortunately, the patient died of a perforated peptic ulcer after 2 months.

Patients receiving immunosuppressive therapy are more likely to develop this condition. For diagnosis, AFB, culture in Lowenstein Jensen medium, and polymerase chain reaction in urine can be used^2^. However, a positive urine AFB test is not diagnostic for tuberculosis since non-tuberculous mycobacteria may be present; moreover, its sensitivity is limited, approximately 40%^3^.

Written informed consent was obtained from the patient for publishing this material.
